# Desorption Electrospray Ionization Mass Spectrometry Reveals Lipid Metabolism of Individual Oocytes and Embryos

**DOI:** 10.1371/journal.pone.0074981

**Published:** 2013-09-20

**Authors:** Andrés Felipe González-Serrano, Valentina Pirro, Christina R. Ferreira, Paolo Oliveri, Livia S. Eberlin, Julia Heinzmann, Andrea Lucas-Hahn, Heiner Niemann, Robert Graham Cooks

**Affiliations:** 1 Institute of Farm Animal Genetics, Friedrich-Loeffler-Institut, Neustadt, Germany; 2 Department of Chemistry, University of Turin, Turin, Italy; 3 Department of Chemistry, Purdue University, West Lafayette, Indiana, United States of America; 4 Department of Pharmacy, University of Genoa, Genoa, Italy; California Institute of Technology, United States of America

## Abstract

Alteration of maternal lipid metabolism early in development has been shown to trigger obesity, insulin resistance, type 2 diabetes and cardiovascular diseases later in life in humans and animal models. Here, we set out to determine (i) lipid composition dynamics in single oocytes and preimplantation embryos by high mass resolution desorption electrospray ionization mass spectrometry (DESI-MS), using the bovine species as biological model, (ii) the metabolically most relevant lipid compounds by multivariate data analysis and (iii) lipid upstream metabolism by quantitative real-time PCR (qRT-PCR) analysis of several target genes (ACAT1, CPT 1b, FASN, SREBP1 and SCAP). Bovine oocytes and blastocysts were individually analyzed by DESI-MS in both positive and negative ion modes, without lipid extraction and under ambient conditions, and were profiled for free fatty acids (FFA), phospholipids (PL), cholesterol-related molecules, and triacylglycerols (TAG). Principal component analysis (PCA) and linear discriminant analysis (LDA), performed for the first time on DESI-MS fused data, allowed unequivocal discrimination between oocytes and blastocysts based on specific lipid profiles. This analytical approach resulted in broad and detailed lipid annotation of single oocytes and blastocysts. Results of DESI-MS and transcript regulation analysis demonstrate that blastocysts produced *in vitro* and their *in vivo* counterparts differed significantly in the homeostasis of cholesterol and FFA metabolism. These results should assist in the production of viable and healthy embryos by elucidating *in vivo* embryonic lipid metabolism.

## Introduction

The lipid composition of oocytes and preimplantation embryos is crucial for mammalian development and heavily affects the success of embryo freezing in animal production systems. Indeed, in animal reproduction, cryopreservation of oocytes and preimplantation embryos facilitates storage of valuable animal genetic resources and in humans it allows infertile couples to have children [1-4].

Free fatty acids (FFA) are stored as triacylglycerols (TAG); uncharged esters of glycerol arranged as virtually anhydrous cytoplasmic droplets [5] that represent a compact energy reserve [6]. Cholesterol (Chol) and phospholipids (PL) are essential for the formation of cellular membranes and are critically important for cell division after fertilization. However, the lipid abundance in oocytes and embryos seems to be species-specific [7]: porcines show the highest oocyte and embryo lipid content of livestock species, followed by bovines and ovines [6]; mice have the lowest lipid content among animal models [8]. The bovine species has been long accepted as a useful model for humans to study the effects of assisted reproductive techniques (ARTs) [9-11]. Due to ethical constraints, only human oocytes that had failed in fertilization attempts have been analyzed and showed relative low total lipid content [12]. Although the lipid content of bovine oocytes and preimplantation embryos differs from that of the human species, clear similarities in reproductive dynamics exist (i.e. time of blastocyst formation, number of ovulated oocytes per estrous cycle, follicular growing pattern, etc.) that render the bovine species a reliable animal model for studying fertility impairments associated with metabolic disorders in humans [5,13-17].

Various attempts have been made to increase our understanding of lipid metabolism during preimplantation embryonic development and to develop strategies to avoid harmful effects associated to *in vitro* culture, including supplementation of specific FFA in diets [18-20] or addition of different types and combinations of FFA to *in vitro* culture media [21-23]. In humans, as in various animal models, metabolic diseases have been associated with assisted reproductive technologies [24] and with particular features of the maternal lipid metabolism [25]. For example, in the case of *in vitro* culture, TAG droplets and FFA accumulate intracellularly which in turn is associated with significant damage of cellular organelles such as mitochondria and the endoplasmic reticulum, thus impairing survival of oocytes and embryos after freezing [26]. However, the molecular mechanisms underlying altered lipid metabolism during embryo *in vitro* culture in comparison to *in vivo* embryo development remain an enigma.

Free fatty acids have been identified in mouse, bovine, pig, sheep and human oocytes and preimplantation embryos by gas-chromatography (GC) and thin-layer chromatography (TLC). These techniques require a large number of individual samples to obtain reliable results [6]. Lipid droplet staining, an alternative technique used for total lipid quantification, requires time-consuming sample preparation and provides no structural information on the detected lipids [4]. Recent progress in mass spectrometry (MS) using matrix-assisted laser desorption/ionization mass spectrometry (MALDI-MS) and desorption electrospray ionization mass spectrometry (DESI-MS) has allowed for the first time resolved, detailed structural analysis of FFA, PL and TAG species present in single oocytes and preimplantation embryos [27-29]. Analysis of large biological datasets like this is facilitated by principal component analysis (PCA), commonly used for exploratory investigation of mass spectral datasets [30]. It allows compression of the data for in-depth characterization of biological samples and has been previously used to analyze DESI-MS data obtained from mouse preimplantation embryos [28].

Gene expression analysis by quantitative real-time PCR (qRT-PCR) has been successfully used to study oocyte and preimplantation embryonic metabolism in relationship to *in vitro* culture [31-33]. Expression of FA synthase (FASN) was significantly reduced in blastocysts from obese mice, and was associated to changes in Chol synthesis regulation, indicating the physiological importance of enzymes and signaling molecules involved in FA and Chol metabolism in postnatal cellular programming [34]. Moreover, recent findings revealed marked differences in the regulation of Chol biosynthesis, sterol synthesis, and cell differentiation when bovine embryos produced *in vivo* were compared to their *in vitro* derived counterparts [35].

Here, we used the bovine model and introduced an analytical approach to gain a better understanding of the complex effects of *in vitro* culture on embryonic metabolism. A broad lipid structural survey of individual oocytes and blastocysts was obtained by DESI-MS, followed by a robust multivariate analysis, using PCA and LDA, with a data fusion strategy applied for the first time to pure MS data. Important differences between the experimental groups were observed in FFA metabolism and Chol biosynthesis. This information was used to design a qRT-PCR analysis to unravel the relative abundance of genes critically involved in lipid metabolism. Finally, MS and qRT-PCR outcomes were comprehensively evaluated providing novel insights into the metabolic implications based on the molecular data.

## Results and Discussion

### Representative DESI-mass spectra and abundant lipid species

**Figure 1:**
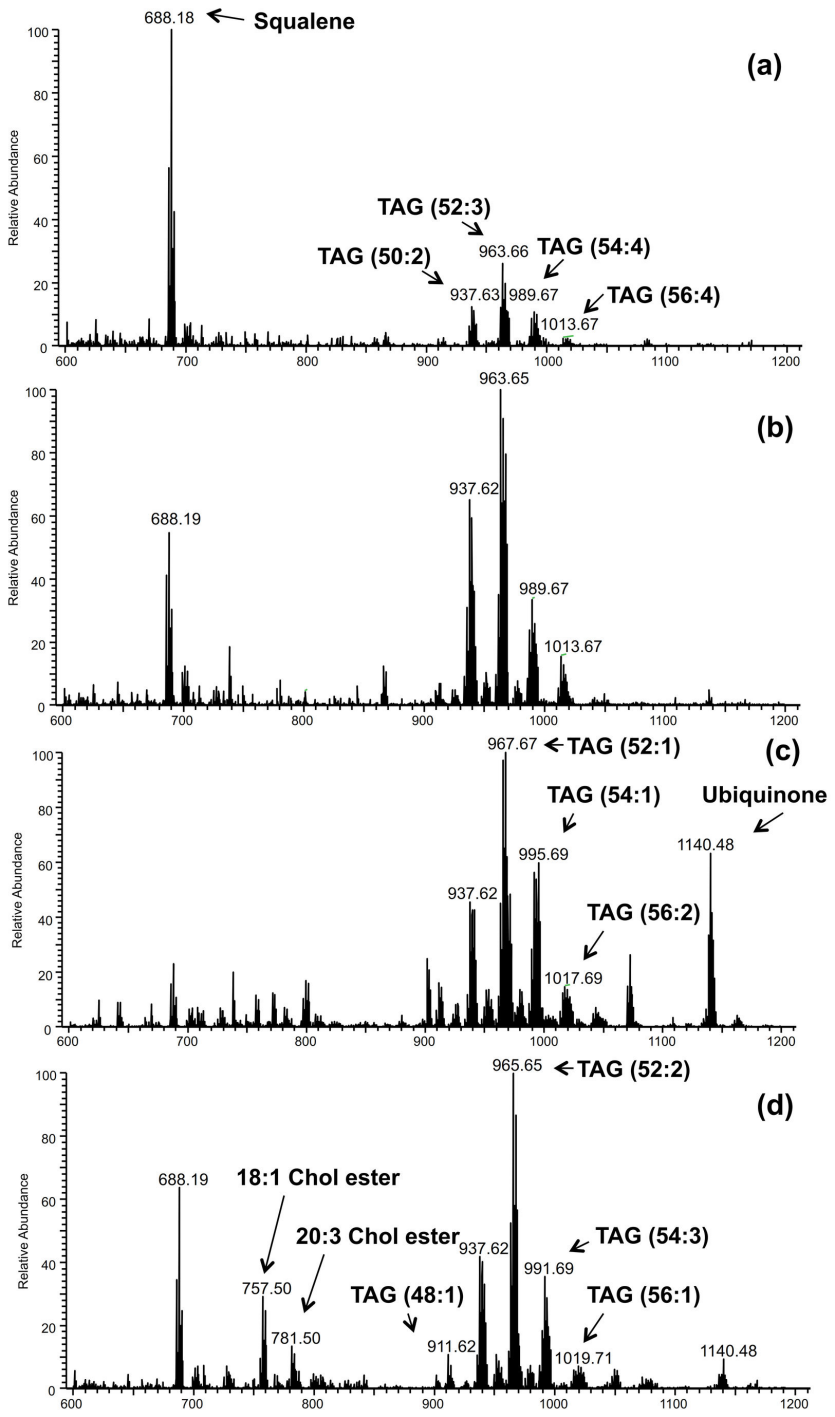
Representative high resolution DESI-MS mass spectra in the positive ion mode. (**a**) immature oocyte; (**b**) *in* *vitro* matured oocyte; (**c**) blastocyst produced *in* *vitro*; (**d**) blastocyst produced *in* *vivo*. See text for basis for tentative lipid class assignments of the major peaks.

Single bovine oocytes and embryos were subjected to DESI-MS analysis in the positive and negative ion modes. Four different developmental stages were analyzed: immature and *in vitro* matured oocytes; blastocysts produced *in vitro* and *in vivo*. Attribution of the lipid species was made using high mass resolution DESI-MS analysis and collision-induced dissociation (CID) tandem MS experiments. Due to the large amount of chemical information obtained by DESI-MS, many lipid ions of low intensity are present in the mass spectra. Here, we report the identification of the most abundant ions. In the positive ion mode, DESI-MS lipid profiles predominantly displayed cytosolic lipids, such as cholesteryl esters (CE) and TAG, besides squalene (*m/z* 688.1) and ubiquinone (*m/z* 1140.4) (see lipid assignments in Table S1 in File S1). The CE species were detected in the *m/z* range of 700-850 and were observed most abundantly in the DESI-mass spectra obtained from blastocysts (Figure 1). The CE species had 16 to 24 carbons and one to six units of unsaturation (Table S1 in File S1). Oleic (18:1) and linoleic (18:2) CE of *m/z* 757.5 and *m/z* 755.4, respectively, gave the most intense CE ions. TAG species containing 48 to 56 carbons and one to five units of unsaturation in the fatty acyl residues, such as TAG (48:1), (48:2), (50:1), (50:2), (52:3), (52:2) predominated in the *m/z* range of 900-1100 (Table S1 in File S1). Some of these TAG species had been previously detected by MALDI-MS analysis of bovine oocytes, but had not been seen in bovine blastocysts, probably due to the ion suppression caused by the PC species in the MALDI analysis performed in the positive ion mode using 2,5-dihydroxybenzoic acid (DHB) as the organic matrix [29]. Free fatty acids, as well as membrane lipids such as phosphatidylethanolamine (PE), phosphoatidylserine (PS), phosphatidylglycerol (PG) and phosphatidylinositol (PI) were the prevalent species detected in DESI mass spectra in the negative ion mode (see lipid assignments in Table S2in File S1). Most abundant FFA present were palmitic (*m/z* 255.2), oleic (*m/z* 281.2), stearic (*m/z* 283.2) and arachidonic (*m/z* 303.2) FA. The most abundant phospholipid (PL) species in the DESI-MS spectra were [PG (34:1)] of *m/z* 747.5, [PS (36:1)] of *m/z* 788.5, PI with an alkyl ether substituent, [PIo (34:1)] of *m/z* 821.5, [PI (36:2)] of *m/z* 861.5, [PI (36:1)] of *m/z* 863.5 and [PI (38:4)] of *m/z* 885.5 (Figure S1).

### Differences in lipid signatures clarified by multivariate analysis

The lipid profiles obtained allow for relative quantification of the detected molecular ions based on their intensity changes without use of internal standards. Diverse DESI-MS applications, including cancer diagnosis [36] and grading of human brain cancer tissue [37] have used this analytical strategy and proved it to be highly reproducible, although only relatively quantitative. Nevertheless, relative quantification by DESI-MS has provided good agreement with LC-MS/MS data, which includes the use of internal standards for absolute quantification [38,39].

The multivariate approach used in this work, PCA, provides a global picture of the chemical information embodied in the mass spectrometric data obtained in both the positive and the negative ion modes.

The lipids that best characterize blastocysts and oocytes in the positive ion mode are unsaturated TAG and CE (Figure S2). These compounds are easily recognized by the characteristic isotopic pattern of silver due to the addition of silver nitrate to the DESI spray solution [40,41]. As observed in the representative mass spectra shown in Figure 1, the most abundant silver-adduct ions for oocytes were *m/z* 963.7/965.7 (TAG 52:3) and *m/z* 965.7/967.7 (TAG 52:2), whereas *m/z* 993.7/995.7 (TAG 54:2), *m/z* 757.5/759.5 (18:1 cholesteryl ester), and *m/z* 686.2/688.2 (squalene) were more abundant in blastocysts.

In the negative ion mode, the ions that best characterize blastocyst samples were of *m/z* 281.2 (oleic acid), which was more abundant in blastocysts produced *in vivo*; *m/z* 255.2 (palmitic acid) and *m/z* 283.2 (stearic acid) were more abundant in *in vitro* derived blastocysts than in their *in vivo* derived counterparts. Conversely, *m/z* 747.5 [PG (34:1)], *m/z* 804.6 [PS (37: 0)], *m/z* 821.6 [PIo (34:1)] and *m/z* 883.5 [PI (38:5)] were present in higher relative abundance in oocytes, independent of the maturation state (Figure S1 and Figure S3).

**Figure 2:**
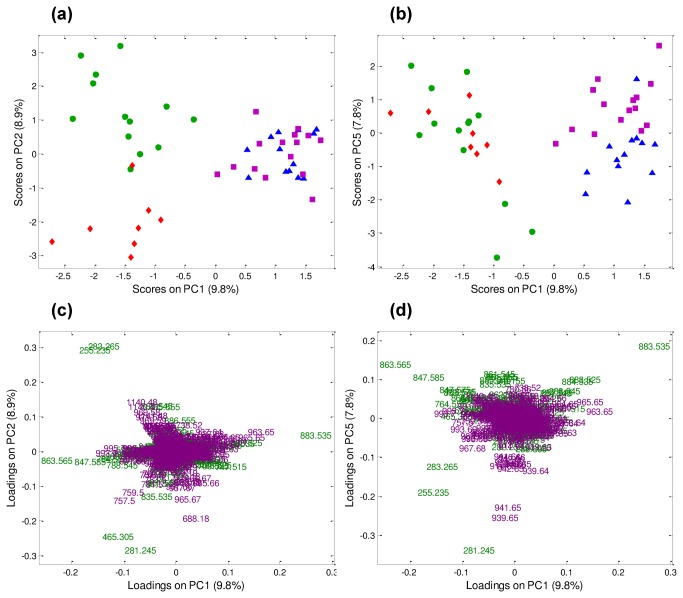
PCA of the fused datasets from positive and negative ion mode mass spectra. (**a**) PC1 vs. PC2 score plot. (**b**) PC1 vs. PC5 score plot. (**c**-**d**) PC1 vs. PC2 (left side) and PC1 vs. PC5 (right side) original loading plots labeled in terms of *m/z* ratio (green: original negative ion mode dataset; violet: original positive ion mode dataset). Blastocysts produced *in* *vitro* (green circles, n= 13), blastocysts produced *in* *vivo* (red diamonds, n= 8), immature oocytes (blue triangles, n= 13) and *in* *vitro* matured oocytes (violet squares, n= 15).

When PCA was performed for the first time by combining both datasets (positive and negative ion modes) using a data fusion strategy (DF-PCA), a clear separation of the four categories was obtained (Figure 2), confirming the synergistic effect of simultaneously considering two independent chemical profiles for sample characterization [42]. Separation between oocytes and blastocysts was visible along PC1 (i.e. highest source of data variability). Moreover, a separation was evident along PC2 for blastocysts produced *in vivo* and *in vitro* (Figure 2a). Differences between immature and *in vitro* matured oocytes were evident along PC5, with only a small contribution to the total data variability (Figure 2b). According to the loading plot, the ions most involved in defining PC1, which separates oocytes and blastocysts, were of *m/z* 863.6 [PI (36:1)], *m/z* 883.6 [PI (38:5)], *m/z* 963.7 [TAG (52:3)], *m/z* 965.7 [TAG (52:2)] and *m/z* 993.7 [TAG (54:2)]; whereas, the main contribution for separating blastocysts *in vivo* and *in vitro* along PC2 was due to ions of *m/z* 281.2 (oleic acid), *m/z* 465.3 (cholesterol sulphate), *m/z* 283.2 (stearic acid), *m/z* 255.2 (palmitic acid), and *m/z* 1140.5 (ubiquinone). Along PC5, *m/z* 963.7 [TAG (52:3)], *m/z* 965.7 [TAG (52:2)], *m/z* 883.5 [PI (38:5)] and *m/z* 939.7 [TAG (50:1)] were important for separating immature and oocytes matured *in vitro* (Figure 2c-d).

**Figure 3:**
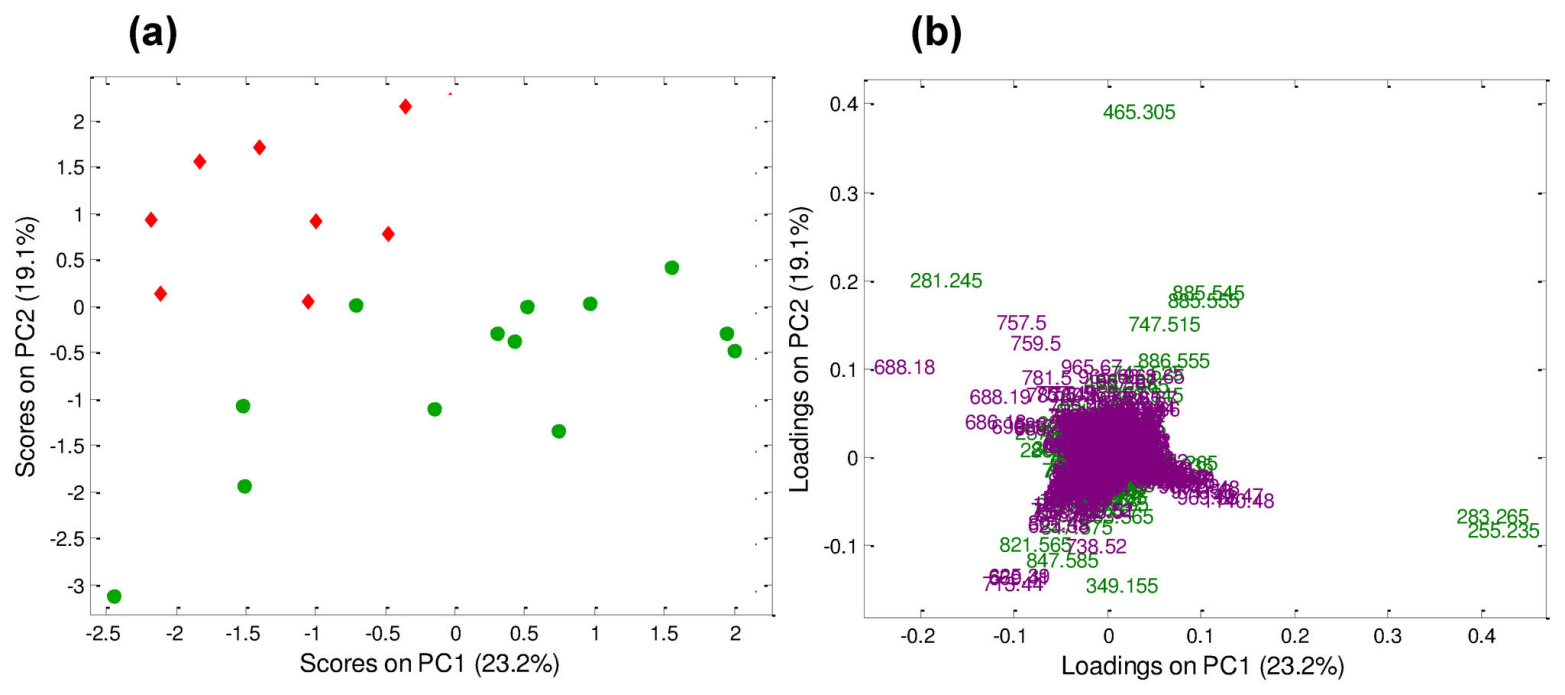
DF-PCA considering only blastocysts. (**a**) PC1 vs. PC2 score plot. Green circles: blastocysts *in* *vitro* (n= 13); red diamonds: blastocysts *in* *vivo* (n= 8). (**b**) PC1 vs. PC2 loading plot labeled in terms of *m/z* ratio (green: negative ion mode; violet: positive ion mode).

DF-PCA was then performed again, considering only immature and *in vitro* matured oocytes, in order to better elucidate the differences between these two groups (Figure S4). According to the loading plot, three species of PI (38:5, 34:1 and alkylated 34:1) in the negative ion mode and the polyunsaturated TAG (52:2) in the positive ion mode, were more abundant in *in vitro* matured than in immature oocytes, in which squalene was present at higher levels. These results are in agreement with differences observed in the representative mass spectra shown in Figure 1 (positive ion mode) and Figure S1 (negative ion mode). Additionally, DF-PCA revealed that in blastocysts produced *in vivo*, oleic acid, cholesterol sulphate, cholesteryl esters of palmitic and eicosatrienoic acids, TAG (52:2) and squalene were more abundant compared to *in vitro* produced blastocysts, in which ions of *m/z* 255.2 (palmitic acid) and *m/z* 283.2 (stearic acid) were prevalent. The DF-PCA analysis considering exclusively blastocysts produced *in vitro* or collected *in vivo* is shown in Figure 3, where PC1 vs. PC2 score and loading plots are shown. Separation between *in vitro* and *in vivo* samples goes diagonally through PC1 vs. PC2 score space. These results are in agreement with differences observed in the representative mass spectra shown in Figure 1 (positive ion mode) and Figure S1 (negative ion mode).

Remarkably, this DF-PCA strategy, which has been employed to combine DESI-MS data obtained in the positive and the negative ion modes, allowed the combination of different datasets collected from the same samples for a common analysis, so as to improve extraction of chemical information useful for sample characterization and/or classification. This strategy provided a novel insight into interactions between cholesteryl esters and TAG with FFA and phospholipids profiles in oocytes and blastocysts, respectively. Moreover, DF-PCA is useful for an unsupervised compression of the fused data, allowing subsequent classification tools to be applied.

### Classification of developmental phase by LDA

LDA was performed to classify samples according to their developmental stage based on chemical information obtained by DESI-MS. Prediction performance was validated using a cross-validation (CV) strategy (Table S3 in File S1). LDA was first performed using the positive ion mode data (dataset of 49 samples and 60001 variables) with 10 CV deletion groups and selecting the first 10 PCs to provide a CV global prediction rate of 79.6%. CV prediction rates for the individual categories were: 100% for blastocysts produced *in vitro* (i.e. all samples assigned to class “*in vitro* Bla” belong to that class), 100% for blastocysts *in vivo*, 61.5% for immature oocytes, and 68.8% for oocytes matured *in vitro* (Table S3 in File S1). When LDA was performed on negative ion mode mass spectra (dataset of 49 samples and 650001 variables), the CV global prediction rate was 89.8% (Table S3 in File S1). In the case of DF-PCA, the LDA strategy leads to 95.9% correct classification (Table S3 in File S1). Although the number of samples is relatively small, the enhancement of the prediction success demonstrates the benefit that data fusion strategy of complementary positive and negative ion mode mass spectra provides to sample characterization and classification. Indeed, the CV confusion matrix for the fused data shows a lower number of falsely assigned samples, consistent with the best results in classification (Table S3 in File S1). The information provided by LDA confirms previous findings of PCA analysis in this work as well as previous reports of MS analysis of individual oocytes and preimplantation embryos [27-29]. These results illustrate the strengths of this analytical approach, which can be applied whenever complementary mass-spectral profiles can be acquired from the same samples to characterize and classify them. Remarkably, ambient ionization MS techniques that allow intact samples to be non-destructively analyzed [43] - without any sample pretreatment steps - represent ideal analytical techniques for data fusion strategies.

### Upstream analysis of DESI-MS lipid profile differences evaluated by qRT-PCR

**Figure 4:**
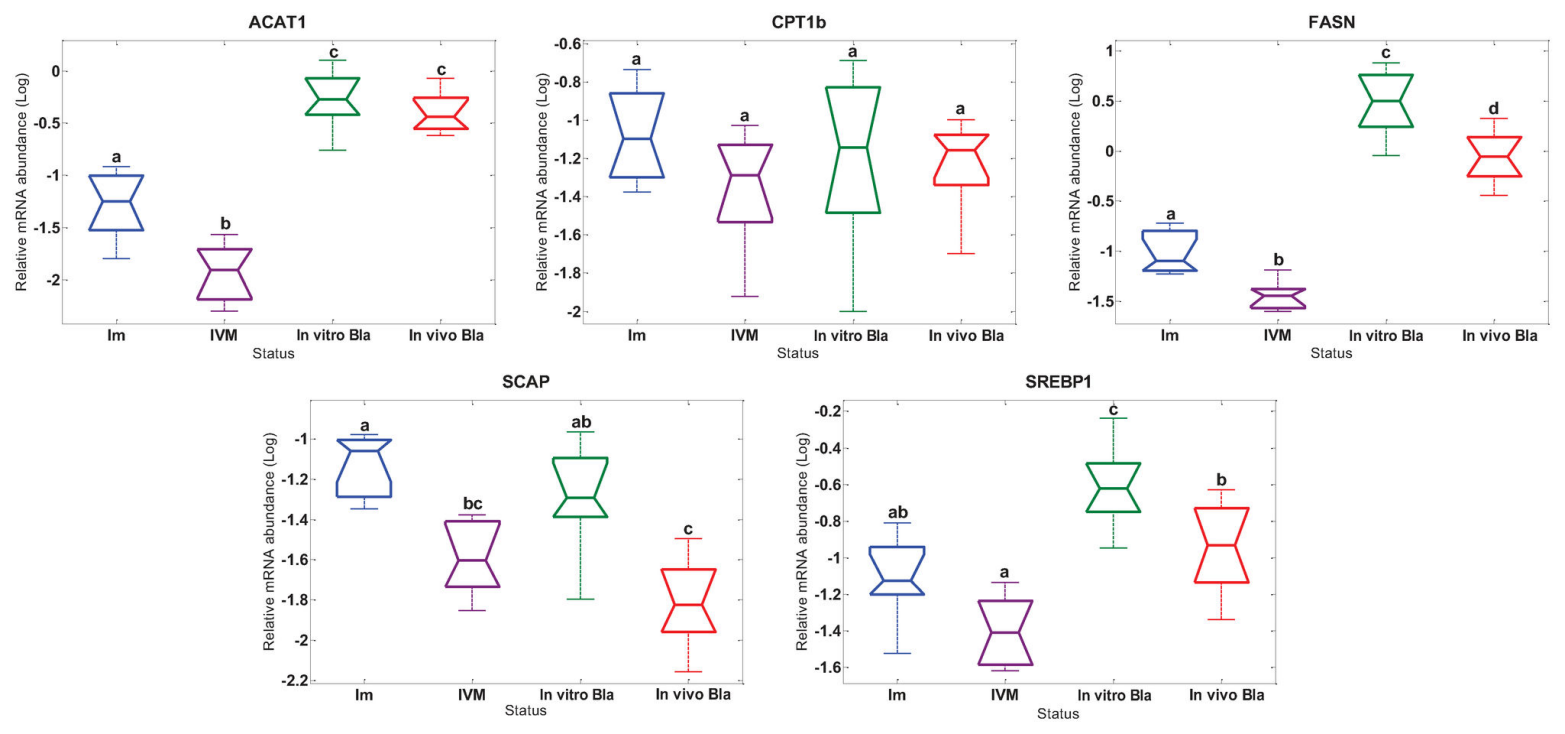
Relative poly (A) + mRNA abundance of target genes. Box plot of Log converted data (with median, 25th and 75th quartiles and max and mix values) based on single oocytes/embryos preparation: immature oocytes, n=8 (Im); *in* *vitro* matured oocytes, n=8 (IVM); *in* *vitro* produced blastocysts, n=7 (*In* *vitro* Bla); *in* *vivo* produced blastocysts, n=8 (*In* *vivo* Bla). Different superscripts indicate significant differences (P< 0.05).

To gain further insight into lipid metabolism, levels of mRNA abundance of genes related to FFA biosynthesis and intracellular Chol homeostasis were examined in individual bovine immature, *in vitro* matured oocytes and blastocysts collected *in vivo* and *in vitro* (Figure 4). The mRNA abundance of Chol acyl transferase (ACAT1), FA synthase (FASN), and SREBP activating protein (SCAP) was significantly (p<0.05) down-regulated in *in vitro* matured oocytes compared to immature oocytes. Moreover, a trend of decreasing mRNA expression of sterol regulatory element binding protein (SREBP1) was observed in oocytes matured *in vitro* when compared with the immature group (P=0.06). SCAP serves as a sterol sensor by activating Site-1 protease, which in turn activates SREBP1 and 2, triggering the activation of genes encoding enzymes of Chol and FA biosynthesis in case of low intracellular sterol levels [34,35,44]. Interestingly, up-regulation of transcripts encoding ACAT1, FASN (dramatically over-expressed), SCAP and SREBP1 was observed when blastocysts produced *in vitro* were compared to their *in vivo* derived counterparts (P<0.05). Transcript abundance of carnitine palmitoyltransferase-1 (CPT 1b) was not altered among the four analyzed groups. A graphical summary of the gene expression results is shown in Figure 4.

### Comprehensive interpretation of DESI-MS and qRT-PCR results

Biochemical information present in mass spectra and the use of a multivariate analysis, such as DF-PCA and LDA, in conjunction with gene expression data allowed comprehensive analysis of lipid metabolism.

We observed characteristic differences in lipid profiles between *in vitro* and *in vivo* produced blastocysts. Specifically, saturated FFA, palmitic acid and stearic acid, were present in much higher relative abundance within *in vitro* produced blastocysts than in their *in vivo* derived counterparts. This observation is in accordance with literature, as palmitic acid had been reported to be the most abundant fatty acid in bovine oocytes, followed by oleic acid, stearic acid and linoleic acid [6]. Linoleic acid is the major fatty acid in bovine follicular fluid, followed by palmitic acid [45,46]. Recent work on the mouse model reported that exposure of cumulus-oocyte complexes (COCs) to palmitic acid at a concentration in the upper range of physiological levels induced endoplasmic reticulum stress, which in turn reduced protein secretion, disrupted mitochondrial activity in oocytes, and impaired oocyte maturation and fertilization [47]. Cell proliferation and the number of inner mass cells in mice blastocysts were reduced concomitantly with an increase in trophoblast cell apoptosis after *in vitro* culture in medium supplemented with 200 µM of palmitic acid [48]. Moreover, supplementation of the culture medium with stearic and palmitic acid during bovine oocyte *in vitro* maturation impaired post-fertilization development in a dose-dependent manner [49]. Similarly, supplementation of culture medium during *in vitro* maturation of bovine oocytes with oleic acid, palmitic acid and stearic acid altered gene expression in blastocysts and reduced embryo quality, based on energy and amino acid metabolism [22]. Presumably, the higher concentrations of FFA within *in vitro* produced blastocysts are responsible for impaired embryonic development. A recent publication has demonstrated alteration of the REDOX status of bovine oocytes and embryos when high levels of free fatty acids were incorporated to the culture media [50].

*In vivo* produced blastocysts showed higher concentrations of neutral lipid species, including cholesterol sulphate, cholesteryl esters of palmitic and eicosatrienoic acids, TAG (52:2), squalene and oleic acid. The accumulation of neutral lipid species, which is the normal energy storage of mammalian cells, may potentially contribute to normal developmental rates of mammalian blastocysts produced *in vivo*. In more detail, squalene, a long-chain poly-unsaturated fatty acid [51], in combination with TAG and sterol esters, has been involved in intracellular lipid droplet formation, clustering in both yeast and mammalian cells [52], probably by regulating membrane properties [53]. To our knowledge, this is the first report of the presence of squalene in mammalian oocytes and preimplantation embryos. Oleic acid is a mono-unsaturated fatty acid proven to be beneficial in bovine post-fertilization development, lipid storage and oocyte maturation [49].

Gene expression analysis was designed based on the DF-PCA analysis of DESI-MS results and genes were selected for this study that encode functional enzymes critically involved in intracellular cholesterol level regulation (SREBP1 and SCAP), long-chain fatty acid biosynthesis (FASN), reversible formation of acetoacyl-CoA from two molecules of acetyl-Coa (ACAT1) and control of the long-chain fatty acid beta-oxidation (CPT 1b). Four of the target genes (SREBP1, FASN, SCAP and ACAT1) were significantly down-regulated after *in vitro* maturation which is in agreement with previous reports, where the relative abundances of mRNAs of genes crucial for oocyte development (SLC2A8, GDF9, PRDX1 and ZAR1) were down-regulated after *in vitro* maturation of bovine oocytes [31,32]. Our results indicate that biosynthesis and storage of neutral lipid species, present in higher levels in *in vitro* matured oocytes than in immature oocytes (PI and TAG of polyunsaturated fatty acids), take place at the acquisition of oocyte developmental competence during folliculogenesis [54]. The presence of high levels of saturated FFA (most likely stearic and palmitic acid) in *in vitro* produced blastocysts detected by DESI-MS coincides with a significant up-regulation of target genes (SREBP1, FASN, SCAP and ACAT1). Fatty acid synthase, which was over-expressed in *in vitro* produced blastocysts, is the most relevant enzyme in the biosynthesis of long-chain fatty acids. Palmitic acid is the main product of FASN by the novo synthesis from Acetyl-CoA, Malonyl-CoA and NADPH [55]. The sterol synthesis sensor SREBP/SCAP was clearly activated in *in vitro* produced blastocysts, which was confirmed by our gene expression results. In agreement with this, a recent publication showed significant up-regulation of 11 genes involved in cholesterol biosynthesis in bovine *in vitro* compared to the *in vivo* produced embryos [35]. In contrast to the present and previous results, genes involved in cholesterol metabolism (HSD17B7, CYP11A1), steroid metabolism (HSD3B1, CYP11A1, APOA1), lipid metabolism (MSMO1, ANXA1, ANXA2) and lipid excretion and translocation (ABCC2) were significantly down-regulated in bovine *in vitro* produced blastocysts in comparison to their *in vivo* counterparts [56].

## Conclusions

This bioanalytical strategy made possible the biological interpretation of DESI and qRT-PCR data for studying the impact of *in vitro* culture on individual bovine oocytes and blastocysts. Here we provide for the first time a comprehensive and in-depth analysis of the lipid composition and its modulation by *in vitro* production methods in both the female gamete (oocyte) and the blastocyst. The analysis of two different datasets (i.e. positive and negative ion mode MS) obtained by state-of-the-art ambient MS from single oocytes or blastocysts, followed by application of multivariate statistical analysis and data fusion, makes this work unique and provides new insights into the complex regulation of mammalian oocyte/embryo lipid metabolism, which can be exported to other mammalian species, including humans. Our results demonstrate that current *in vitro* production systems are associated with profound alterations of the lipid profile and metabolism of preimplantation embryos with possibly deleterious effects on development and health status of the offspring. Using this novel analytical approach to study upstream lipid metabolism, our findings pave the way for monitoring and improvement of *in vitro* culture systems and reproductive techniques. Further, this work might contribute towards a better understanding of the concept of the embryonic onset of adult disease [57,58] and metabolic impairments associated to lipid metabolism such as obesity and diabetes type II.

## Materials and Methods

### Cumulus-oocyte complexes (COCs) recovery, *in vitro* maturation and blastocysts production *in vitro*

Bovine ovaries were collected from a local abattoir (Westfleisch Lübbecke, Westfalen, Germany) after approval by the local supervisory veterinarian. The ovaries were transported to the host laboratory (Mariensee, Germany) in saline solution (0.9%) at 30°C supplemented with penicillin and streptomycin sulphate. After cumulus cells removal, immature oocytes were stored as described below for DESI-MS analysis. Another group included *in vitro* matured oocytes; a further subset was used for blastocyst production *in vitro* [59,60]. Single oocytes (immature, n=8 and *in vitro* matured, n=8) and blastocysts (produced *in vivo*, n=8 and produced *in vitro*, n=7) were frozen in a minimum volume (≤ 5 µL) of PBS supplemented with 1% polyvinyl alcohol (PVA) medium in a 600 µL siliconized cryovials (Biozym Diagnostic GmbH, Hessisch Oldendorf, Germany) and stored at −80 °C until analysis (an extended version of these methods can be found in File S2). A separate ethical approval is not necessary for *in vitro* experiments with bovine oocytes/embryos in Germany. All experiments were performed in strict accordance with the United States and Germany’ laws and guidelines and approved by institutional committees [61], specifically the guidelines on good scientific practice from DFG (Deutsche Forschungsgemeinschaft).

### *In vivo* embryo production

Dairy cows from the Institute’s experimental herd in Mariensee (Germany) were super-ovulated between 9 and 12 days of the estrous cycle by using intramuscular injections of 2500-3000 IU eCG (Intergonan®; Intervet, Tönisvorst, Germany) and Cloprostenol (Estrumate®; Essex, Munich, Germany) 48 h later. After 48 h, cows were inseminated twice with an interval of 12 h. Eight days later, blastocysts were collected by nonsurgical uterine flushing with 500 mL PBS supplemented with 1% NBCS (newborn calf serum, Invitrogen, Karlsruhe, Germany) [62]. The blastocysts were washed three times in PBS with 0.1% PVA under a stereomicroscope. Single embryos were frozen in individual cups at −80 °C and stored until further analysis.

### DESI-MS analysis and attribution of most abundant lipid species of high resolution mass spectrometry

Samples were stored in minimal volume (2-5µL) of PBS supplemented with 0.1% polyvinyl alcohol (PVA) and were shipped on dry ice from the Institute of Farm Animal Genetics (Mariensee, Germany) to Purdue University (West Lafayette, IN, USA; USDA permit 118624 Research). Mouse brain tissue sections and part of the samples have been used for DESI system optimization (Purdue University Animal Care and Use Committee approved protocol is No. 1111000314, as reported in [28]).

The samples (n=82) were submitted to lipid profile analysis in the positive ion mode, in order to detect cholesteryl esters and TAG data by doping the solvent spray with silver ions [40,41,43]. The use of silver nitrate in the DESI-MS solvent spray promotes the formation of lipid silver adducts, which are highly ionizable. Due to the silver isotopic distribution, combinations of ions, such as *m/z* 963.7/965.7 (TAG 52:3), correspond to the ^107^Ag and ^109^Ag adducts of the same lipid molecule. Then the same samples were analyzed in the negative ion mode for FFA and PL profiling [27,28]. A total of 49 samples was analyzed in both positive and negative ion modes.

A Thermo Scientific Exactive (San Jose, CA, USA) mass spectrometer was used for the experiments. DESI-MS profiles were acquired in lab-built stage and the DESI spray was positioned ~2 mm from the surface at an incident angle of 50 °. The DESI spray had 5 kV applied to the stainless steel needle syringe and nitrogen gas pressure was 180 psi (Figure A in File S2). Instrument conditions are described in details in the File S2.

Molecular formula matching and error calculations were performed using the instrument software Xcalibur v.1.0.1.03 (Thermo, Fisher Scientific San Jose, CA, USA) and online search of lipids containing the calculated molecular formulae was carried out in the LIPID MAPS database (Figure B in File S2) [63]. Lipid attributions based on high mass resolution mass measurements data are listed in Table S1 in File S1 (positive ion mode) and Table S2 in File S1 (negative ion mode).

### Multivariate data analyses: PCA and LDA

PCA is commonly used for exploratory investigations of the complex information contained in a full mass spectral dataset, to allow simultaneous analysis of all spectral variables and their inter-correlations [30]. Linear discriminant analysis (LDA) can be applied in combination with PCA for sample classification [36]. Moreover, multivariate analysis together with data-fusion allows suitable sample characterization and classification [42]. Considering the 49 samples from which both positive and negative ion mode mass spectra were collected (immature oocytes, n=13; *in vitro* matured oocytes, n=15; blastocysts produced *in vivo*, n=8; blastocysts produced *in vitro*, n=13), PCA was first performed on two separated datasets, as described extensively in File S2. Furthermore, a data fusion strategy (DF-PCA) was applied on the same datasets in order to investigate whether the joined analysis of positive and negative mass spectra improved sample characterization and/or classification [42]. In view of the huge number of variables constituting each mass spectral dataset, a preliminary compression of the individual datasets by means of PCA was performed (as represented in Figure C in File S2) [42]. By applying the same strategy (positive and negative ion mode mass spectra individually considered and thus fused together), LDA was performed as an additional classification method, in order to quantify the ability of DESI-MS lipid profiles in discriminating among oocytes (immature and matured *in vitro*) and blastocysts (produced *in vivo* and *in vitro*). Details are reported in File S2.

Data processing was performed by means of the in-house Matlab (The Math Works, Inc., Natick, USA) routines.

### Determination of the relative abundance of mRNA transcripts for ACAT1, CPT 1b, FASN, SREBP1 and SCAP

Poly (A) + RNA from single oocytes (immature, n=8 and matured *in vitro*, n=8) and blastocysts (produced *in vivo*, n=8 and produced *in vitro*, n=7) was isolated using the Dynabeads® mRNA DIRECT™ kit (Invitrogen, Carlsbad, USA) as reported previously [31,64]. Two µL of the RT reaction were used for real-time PCR amplification (for more details see File S2). Gene expression data (Log-transformed) were analyzed for each single gene by one-way ANOVA using JMP (SAS Institute Inc., Cary, NC, USA). The Tukey-Kramer test was applied for a multiple comparison of means. For all tests, a P-value ≤ 0.05 was considered as statistically significant.

## Supporting Information

File S1 **Supplementary tables containing details about (i) the attribution of the lipid species made using high mass resolution DESI-MS analysis and collision-induced dissociation (CID) tandem MS experiments in positive and negative ion modes (Tables S1 and S2); (ii) CV confusion matrix for all LDAs (Table S3).** Table S1, Positive ion mode mass spectra. Table S2, Negative ion mode mass spectra. Table S3, CV confusion matrix for all LDAs.
(DOCX)

File S2 **Extended version of Material and Methods containing details about (i) DESI-MS conditions for lipid profile detection; (ii) PCA and LDA methodology and relative results on both individual and fused datasets; (ii) RNA extraction and quantitative RT-PCR.**
(DOCX)

Figure S1 **Representative high resolution DESI-MS mass spectra in the negative ion mode.** (**a**) immature oocyte; (**b**) in vitro matured oocyte; (**c**) blastocyst produced in vitro; (**d**) blastocyst produced in vivo. See text and Table S2 in File S1 for tentative lipid class assignments of the major peaks.
(TIF)

Figure S2 **PCA of the positive ion mode mass spectral data.** Blastocysts in vitro (green circles, n=13), blastocysts in vivo (red diamonds, n=8), immature oocytes (blue triangles, n=13) and in vitro matured oocytes (violet squares, n=15). (**a**) PC1 vs. PC4 score plot. (**b**) PC1 vs. PC4 loading plot labeled in terms of m/z ratio.
(TIF)

Figure S3 **PCA of the negative ion mode mass spectral data.** In vitro blastocysts (green circles, n=13), in vivo blastocysts (red diamonds, n=8), immature oocytes (blue triangles, n=13) and in vitro matured oocytes (violet squares, n=15). (**a**) PC1 vs. PC4 score plot. (**b**) PC1 vs. PC4 loading plot labeled in terms of m/z ratio.
(TIF)

Figure S4 **DF-PCA considering only oocytes.** (**a**) PC1 vs. PC5 score plot. Blue triangles: immature oocytes (n=13); violet squares: in vitro matured oocytes (n= 15). (**b**) PC1 vs. PC5 loading plot labeled in terms of m/z ratio (green: negative ion mode; violet: positive ion mode).
(TIF)
