# Supplementary material for: Desorption Electrospray Ionization Mass Spectrometry Reveals Lipid Metabolism of Individual Oocytes and Embryos
Source: PLoS One. 2013 Sep 20;8(9):e74981. doi: 10.1371/journal.pone.0074981 (PMC3779253; doi:10.1371/journal.pone.0074981)
Supplement: File S1 — Supplementary tables containing details about (i) the attribution of the lipid species made using high mass resolution DESI-MS analysis and collision-induced dissociation (CID) tandem MS experiments in positive and negative ion modes (Tables S1 and S2); (ii) CV confusion matrix for all LDAs (Table S3). Table S1, Positive ion mode mass spectra. Table S2, Negative ion mode mass spectra. Table S3, CV confusion matrix for all LDAs. (DOCX) [file pone.0074981.s001.docx]

**Table S1.** **Positive ion mode mass spectra.** Values of *m/z* with high resolution mass accuracy, predicted molecular formula, error in ppm (Delta ppm) of cholesteryl esters and triacylglycerols detected in the positive ion mode as silver adducts by DESI-MS in bovine immature and matured *in vitro* oocytes, and in blastocysts produced *in vitro* and *in vivo*.

| **Ion** | **Molecular formula** | **Delta ppm*** | **Attribution**** |
| --- | --- | --- | --- |
| 688.18760 | C_30_H_50_O_3_NAg_2_ | 0.391 (for *m/z* 686.18803)*** | Squalene |
| 729.47299 | C_43_H_74_O_2_Ag | -0.0054 | 16:1 Cholesteryl ester |
| 753.47308 | C_45_H_74_O_2_Ag | 0.067 | 18:3 Cholesteryl ester |
| 755.48857 | C_45_H_76_O_2_Ag | -0.145 | 18:2 Cholesteryl ester |
| 757.50433 | C_45_H_78_O_2_Ag | 0.0001 | 18:1 Cholesteryl ester |
| 779.48887 | C_47_H_76_O_2_Ag | -0.0968 | 20:4 Cholesteryl ester |
| 781.50426 | C_47_H_78_O_2_Ag | -0.089 | 20:3 Cholesteryl ester |
| 803.48940 | C_49_H_76_O_2_Ag | -0.279 | 22:6 Cholesteryl ester |
| 805.50443 | C_49_H_78_O_2_Ag | -1.048 | 22:5 Cholesteryl ester |
| 813.56707 | C_49_H_86_O_2_Ag | -0.989 | 22:1 Cholesteryl ester |
| 841.59879 | C_51_H_90_O_2_Ag | -0.457 | 24:1 Cholesteryl ester |
| 909.60918 | C_51_H_94_O_6_Ag | -0.009 | TAG (48:2) |
| 911.62441 | C_51_H_96_O_6_Ag | -1.507 | TAG (48:1) |
| 935.62479 | C_53_H_96_O_6_Ag | -1.062 | TAG (50:3) |
| 937.62624 | C_53_H_98_O_6_Ag | -1.230 | TAG (50:2) |
| 939.65531 | C_53_H_100_O_6_Ag | -1.888 | TAG (50:1) |
| 963.65617 | C_55_H_100_O_6_Ag | 0.033 | TAG (52:3) |
| 965.65722 | C_55_H_102_O_6_Ag | -0.131 | TAG (52:2) |
| 987.65590 | C_57_H_100_O_6_Ag | -1.198 | TAG (54:5) |
| 989.67149 | C_57_H_102_O_6_Ag | -1.257 | TAG (54:4) |
| 991.68702 | C_57_H_104_O_6_Ag | -0.422 | TAG (54:3) |
| 993.70316 | C_57_H_106_O_6_Ag | -0.325 | TAG (54:2) |
| 1013.67294 | C_59_H_102_O_6_Ag | -0.684 | TAG (56:4) |
| 1015.68742 | C_59_H_104_O_6_Ag | -0.949 | TAG (56:3) |
| 1017.70422 | C_59_H_106_O_6_Ag | 0.735 | TAG (56:2): |
| 1019.71944 | C_59_H_108_O_6_Ag | -2.239 | TAG (56:1) |
| 1140.47997 | C_59_H_90_O_7_NAg_2_ | -0.612 (for *m/z* 1138.48067)*** | Ubiquinone (Coenzyme Q10) |

*The Delta ppm column displays the difference between the specified mass and the calculated mass in ppm units. High mass resolution attributions have been performed using only *m/z* values for ^107^Ag adducts.

**Attribution of cholesteryl esters and triacylglycerols has been made based on Lipid Maps (www.lipidmaps.org) search of the predicted molecular formulae.

***Due to the fact the squalene and ubiquinone were adducts presenting Ag_2_NO_3_, the exact mass has been calculated based on theses compounds monoisotopic mass. The exact mass matches have been also confirmed by the use of analytical standards and the comparison of isotopic distribution of ions present in samples and analytical standards (Fig. S6).

Abbreviation used: TAG – triacylglycerol.

**Table S2. Negative ion mode mass spectra.** Values of *m/z* with high resolution mass accuracy, predicted molecular formula, error in ppm (Delta ppm) of fatty acids and phospholipids detected in the negative ion mode by DESI-MS in bovine immature and *in vitro* matured oocytes, and in blastocysts produced *in vitro* and *in vivo*.

| **Ion** | **Molecular formula** | **Delta ppm*** | **Attribution**** |
| --- | --- | --- | --- |
| 253.21711 | C_16_H_29_O_2_ | 1.402 | 16:1*** (palmitoleic acid) |
| 255.23275 | C_16_H_31_O_2_ | 1.351 | 16:0 (palmitic acid) |
| 281.24843 | C_18_H_33_O_2_ | 1.333 | 18:1 (oleic acid) |
| 283.26412 | C_18_H_33_O_2_ | 1.463 | 18:0 (stearic acid) |
| 303.23284 | C_20_H_31_O_2_ | 1.434 | 20:4 (arachidonic acid) |
| 465.30418 | C_27_H_45_O_4_S | 0.873 | Cholesterol sulphate |
| 700.52869 | C_39_H_75_NO_7_P | 1.604 | PEp (34:1) |
| 722.51317 | C_41_H_73_NO_7_P | 1.735 | PEp (36:4), PEo (36:5) |
| 742.53940 | C_41_H_77_O_8_NP | 1.709 | PE(36:2) |
| 747.51818 | C_40_H_76_O_10_P | 1.496 | PG(34:1) |
| 764.52352 | C_43_H_75_O_8_NP | 0.642 | PE ( 38:5) |
| 766.53925 | C_43_H_77_O_8_NP | 0.744 | PE (38:4) |
| 760.51297 | C_40_H_75_O_10_NP | 0.867 | PS (34:1) |
| 773.53394 | C_42_H_78_O_10_P | -1.475 | PG (36:2) |
| 784.51357 | C_42_H_75_O_10_NP | 1.606 | PS(36:3) |
| 786.52901 | C_42_H_77_O_10_NP | 1.334 | PS (36:2) |
| 788.54504 | C_42_H_79_O_10_NP | 1.813 | PS (36:1) |
| 804.57592 | C_43_H_83_O_10_NP | 1.255 | PS (37:0) |
| 821.56643 | C_43_H_82_O_12_P | 15.323 | PIo (34:1) |
| 835.53422 | C_43_H_80_O_13_P | 1.334 | PI (34:1) |
| 857.51831 | C_45_H_78_O_13_P | 0.357 | PI(36:4) |
| 859.53453 | C_45_H_80_O_13_P | 1.020 | PI (36:3) |
| 861.55004 | C_45_H_82_O_13_P | 1.491 | PI (36:2) |
| 863.56604 | C_45_H_84_O_13_P | 1.893 | PI (36:1) |
| 883.53403 | C_47_H_80_O_13_P | 1.047 | PI (38:5) |
| 885.54989 | C_47_H_82_O_13_P | 1.281 | PI (38:4) |
| 887.56658 | C_47_H_84_O_13_P | 2.450 | PI (38:3) |

* The Delta ppm column displays the difference between the specified mass and the calculated mass in ppm units.

** Attribution of fatty acids and phospholipids has been made based on Lipid Maps (www.lipidmaps.org) search of the predicted molecular formulae.

***(C:U) represents the number of carbon atoms (C) and the number of unsaturations (U) in the fatty acid chains.

Abbreviations used: PI, phosphatidylinositol; PS, phosphatidylserine; PG, Phosphatidylglycerol, FA, fatty acid. The 'o-' suffix is used to indicate the presence of an alkyl ether substituent, whereas the 'p-' suffix is used for the 1Z-alkenyl ether (plasmalogen) substituent.

**Table S3.** **CV confusion matrix for all LDAs.** Blastocysts produced *in vitro* (*In vitro* Bla, n=13), blastocysts produced *in vivo* (*In vivo* Bla, n=8), immature oocytes (Im, n=13), *in vitro* matured oocytes (IVM, n=15).

| **LDA on positive ion mode mass spectra** | | | | |
| --- | --- | --- | --- | --- |
| **Groups** | ***In vitro* Bla** | ***In vivo* Bla** | **Im** | **IVM** |
| ***In vitro* Bla** | 12 | 0 | 0 | 0 |
| ***In vivo* Bla** | 0 | 8 | 0 | 0 |
| **Im** | 1 | 0 | 8 | 4 |
| **IVM** | 0 | 0 | 5 | 11 |
| **LDA on negative ion mode mass spectra** | | | | |
| ***In vitro* Bla** | 13 | 2 | 0 | 0 |
| ***In vivo* Bla** | 0 | 6 | 0 | 0 |
| **Im** | 0 | 0 | 11 | 1 |
| **IVM** | 0 | 0 | 2 | 14 |
| **LDA on fused data** | | | | |
| ***In vitro* Bla** | 12 | 0 | 0 | 0 |
| ***In vivo* Bla** | 1 | 8 | 0 | 0 |
| **Im** | 0 | 0 | 13 | 1 |
| **IVM** | 0 | 0 | 0 | 14 |
