# Supplementary material for: Desorption Electrospray Ionization Mass Spectrometry Reveals Lipid Metabolism of Individual Oocytes and Embryos
Source: PLoS One. 2013 Sep 20;8(9):e74981. doi: 10.1371/journal.pone.0074981 (PMC3779253; doi:10.1371/journal.pone.0074981)
Supplement: File S2 — Extended version of Material and Methods containing details about (i) DESI-MS conditions for lipid profile detection; (ii) PCA and LDA methodology and relative results on both individual and fused datasets; (ii) RNA extraction and quantitative RT-PCR. (DOCX) [file pone.0074981.s002.docx]

**Supplementary Materials and Methods**


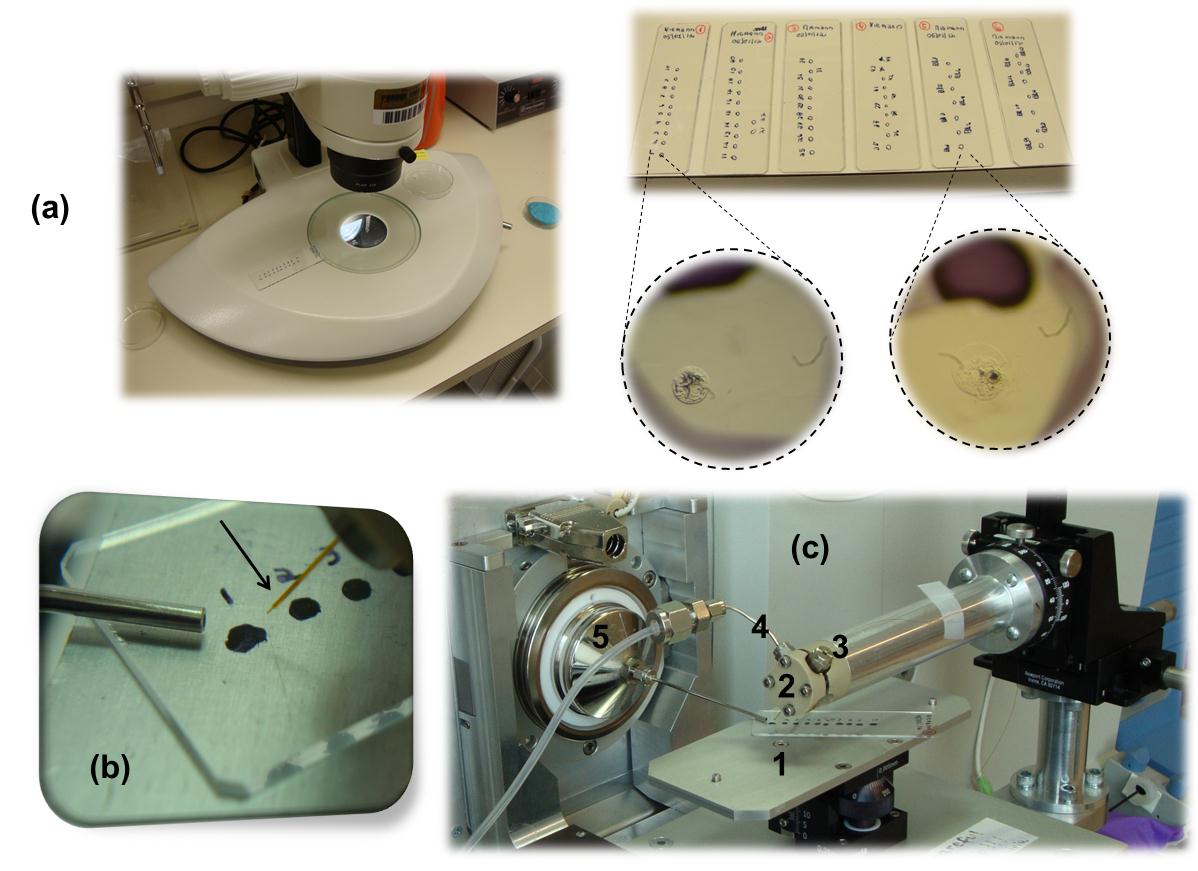


**Figure A.** **DESI-MS analysis of single oocytes and preimplantation embryos workflow.** **(a)** Bovine oocytes and embryos were stored individually and removed from the microtubes by adding ~50 μL of PBS supplemented with 0.1 % PVA and placed in droplets inside disposable 35 mm petri dishes. Samples where then individually placed onto the surface of glass slides (Gold Seal, Portsmouth, NH, USA), which contained small circles drawn in the back with a permanent marker, in order to facilitate the identification and labeling of the samples. After the samples dried completely (around one hour at room temperature), the volume of 1 mL of methanol/ultrapure water 1:1 (v/v) was carefully deposited onto the glass slides and removed by orienting the glass slide vertically. This procedure was needed for removing the PBS salts present in the samples and to avoid the need of individually washing each sample before placing them in the glass slides. Glass slides containing the samples were kept frozen (–80 °C) until shortly before DESI-MS analysis, when they were thawed and allowed to dry at room temperature for 15 min. **(b)** The DESI spray (arrow) was directed to individual oocytes/blastocysts (black background facilitates sample visualization). The metal capillary end is ~2 mm of distance from the sample, in order to collect the lipid ions formed and conduct to the mass spectrometer. **(c)** DESI experimental settings: (1) Glass slide containing the embryos placed under the DESI spray and close to the instrument’s metal capillary, (2) DESI spray support with the (3) solvent entry (inner capillary) and (4) the nitrogen gas entry (outer capillary).

**
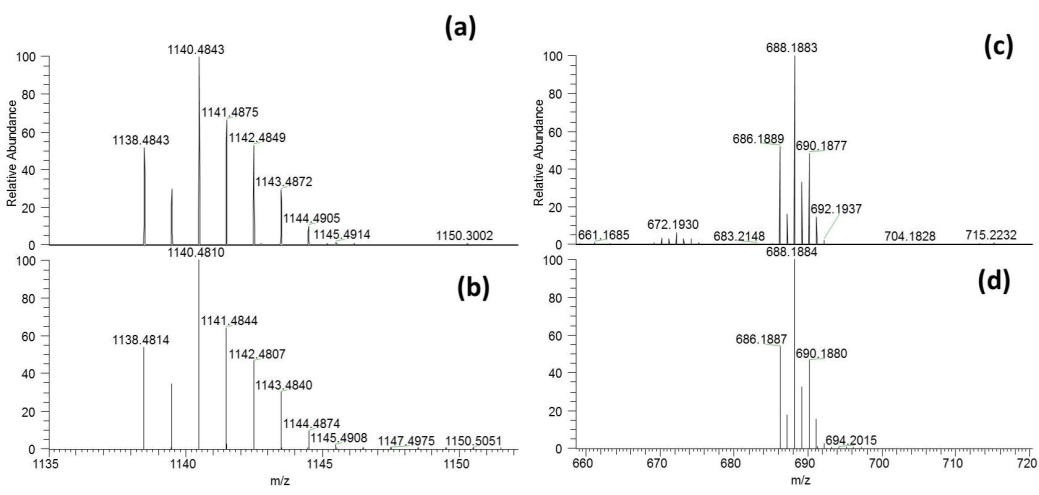
**

**Figure B.** **Attribution of ubiquinone and squalene.** Attribution was based not only on high resolution mass attribution in experimental samples (Table S1), but also on the use of analytical standards to predict formula and isotopic pattern using the Xcalibur software (Thermo Scientific, San Jose, CA, US). **(a)** High mass resolution DESI-MS mass spectrum of the ubiquinone standard showing the region of *m/z* 1135-1155. **(b)** The correspondent theoretical isotopic distribution of the predicted formula C_59_H_90_O_7_NAg_2_ or ubiquinone + Ag_2_NO_3_ (ppm error: 2.568). **(c)** High resolution DESI-MS mass spectrum of the squalene standard showing the region of *m/z* 660-720. **(d)** The correspondent theoretical isotopic distribution of C_30_H_50_O_3_NAg_2_ or squalene + Ag_2_NO_3_ (ppm error: 0.153).

**Figure C.** **Diagram of the DF-PCA strategy used in this study.** **(a)** PCA and LDA were performed on the original datasets structured with R rows (49 samples) and V variables (positive ions: 60001; negative ions: 65001), individually. The same number of PCs (10) was selected for each original dataset. **(b)** PCs scores (S) were merged into a new data matrix; PCA and LDA were performed again.

In vitro oocyte maturation and blastocyst production

Cumulus-oocyte complexes (COCs) were recovered from the ovaries by slicing with a multi-blade knife in Dulbecco’s PBS (Sigma-Aldrich, Munich, Germany) supplemented with 0.33 mM Na-pyruvate, 5.56 mM Glucose, 0.9 mM Calcium Chloride dehydrate, 50 μg/mL streptomycin, 6 μg/mL penicillin G, 4 IU/L Heparin, and 1 mg/mL bovine serum albumin (BSA) (fraction V, Sigma-Aldrich, Munich, Germany). COCs were selected under a stereomicroscope in TCM-air (TCM199, Sigma-Aldrich) supplemented with 50 μg/mL gentamycin sulphate (Sigma-Aldrich), 0.2 Na-pyruvate (Sigma-Aldrich), 4.2 mM NaHCO_3_ (Roth, Karlsruhe, Germany), and 1 mg/mL BSA (Sigma-Aldrich), and only oocytes with at least three layers of compact cumulus cells with a homogeneous granulated cytoplasm were used for further procedures [1]. Maturation medium consisted of TCM 199 at pH 7.4 supplemented with 0.2 mM Napyruvate, 25 mM NaHCO_3_, 50 μg/mL gentamycin, 10 IU/mL eCG, 5 IU/mL of hCG (Suigonan®, Intervet, Tönisvorst, Germany), and 0.1% fatty acid free BSA (Sigma-Aldrich, Munich, Germany). Oocytes were matured in a humidified atmosphere composed of 5% CO_2_ at 39 °C for 24 h under silicone oil. For *in vitro* fertilization, COCs were placed in Fert-TALP containing HHE (10 μM hypotaurine (Sigma-Aldrich), 1 μM epinephrine (Sigma-Aldrich), 0.1 IU/mL heparin (Serva, Heidelberg, Germany)), and 6 mg/mL BSA [2,3]. Frozen semen from a bull of proven fertility was thawed at 30 °C for 25 sec and layered carefully on 1 mL of BoviPureTM 90 % (Labotec, Goettingen, Germany). After that, semen was centrifuged at 400 g for 10 min, followed by removal of the supernatant and re-suspension in 750 μL of fertilization medium (Fert-TALP) containing 6 mg/mL BAS (fraction V) and centrifuged at 400 g for 3 min. This washing step was repeated once using Fert-TALP containing HHE. Finally, the supernatant removed carefully. The final sperm concentration added per 100 μL fertilization drop was 1 × 106 spermcells/mL. COCs and sperm cells were coincubated for 19 hours under silicone oil at 5 % CO_2_ in air at 39 °C. Modified Synthetic oviduct fluid (mSOF) supplemented with BSA-FAF was employed for in vitro culture [4]. Presumptive zygotes were transferred into drops containing 30 μL of mSOF after complete removal of the adhering cumulus cells by repeated pipetting. Embryos were cultured under silicone oil (Serva, Heildelberg, Germany) at 39 °C in a humidified atmosphere composed of 5% CO_2_ and 5 % O_2_ until the expanded blastocyst stage (day 8) [5,6].

Instrumental conditions from DESI-MS

For the DESI-MS experiments in the positive ion mode, acetonitrile (ACN) doped with 5.0 μg/mL with silver nitrate at 4 μL/min flow rate was used. Instrument injection time was 1000 ms, *m/z* range 600-1200, 60 V capillary voltage and 120 V tube lens voltage. The use of AgNO_3_ in the DESI spray allows obtaining lipid silver adducts, which are easily recognized by the characteristic 1:1 abundance ratio of the ^107^Ag:^109^Ag isotopes. For the negative ion mode, solvent combination used was ACN + dimethylformamide (DMF) 1:1 (v/v) at 1.0 μL/min flow rate as already reported [7] and the instrument parameters were: injection time 500 ms, 2 microscans, *m/z* range 150-1000, –50 V capillary voltage and –25 V tube lens voltage. Positive ion mode data were acquired first, followed by acquisition in the negative ion mode. For the data acquisition, the background was recorded and then data of up to 10 samples from experimental group in the same chronogram. Between samples, the glass slide was removed from the DESI spray spot to allow the ion current to go down and provide evident intervals between the background and each sample mass spectra, as shown in Figure D.

**
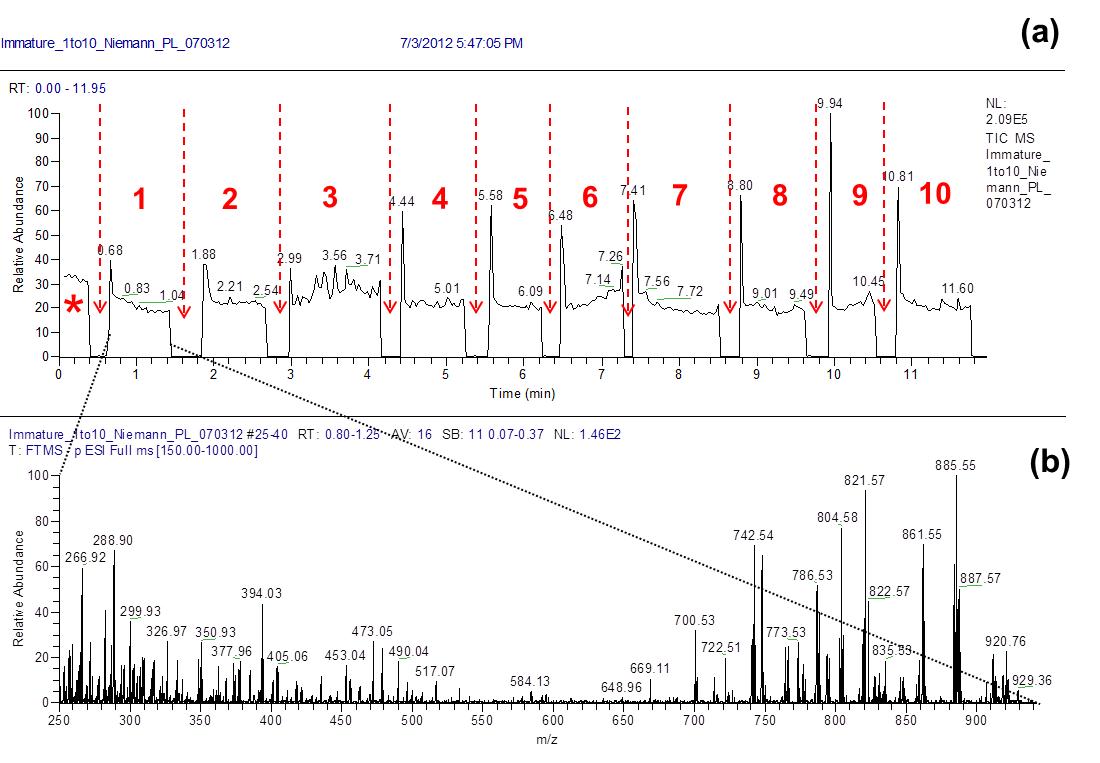
**

**Figure D.** **Exactive mass spectrometer software (XCalibur) output.** **(a)** Chronogram showing the data of ten samples (numbers 1 to 10). For the data acquisition, first the background of the glass slide is collected (*). Then the glass slide is removed and no ion signal is observed (dashed arrows). The numbers 1 to 10 show the ion current during data acquisition for each sample (~1 min/sample). **(b)** Representative negative ion mode mass spectrum of an immature oocyte sample.

Principal component analysis

For each sample, a list of *m/z* values and ion abundances (after background subtraction) from averaged mass spectra was imported into Matlab (The MathWorks, Inc., Natick, USA) and multivariate data processing was performed by means of in-house Matlab routines. For the positive ion mode, the mass range *m/z* 600-1200 was structured with 60001 m/z variables, whereas for the negative ion mode, each representative mass spectrum was structured with 65001 *m/z* variables in the reduced mass range *m/z* 250-900 (thereby excluding uninformative regions of mass spectra). Two PCA were performed on two different data sets (49 rows and 60001 variables; 49 rows and 65001 variables) in order to separately explore the information content of the mass spectra in positive ion mode and negative ion mode, respectively, in characterizing samples according to their developmental stage and *in vivo* vs. *in vitro* condition. PCA was performed on column-centered data, after normalization with respect to the total ion current (TIC), so as to correct signal intensity for instrumental variability. PCA is commonly used for exploratory investigations of the complex information contained in a full mass spectral dataset, to allow consideration of all the spectral variables and their inter-correlations, simultaneously. PCA is capable of re-organizing the information in a dataset of samples, so as to describe the almost-total data variability with considerably fewer variables and, therefore, can be used as strategy for data compression. The principal components (PCs) can be considered as orthogonal (*i.e.* uncorrelated) directions in the multidimensional data space that efficiently describe large fractions of the information. The projections of the data objects (*i.e.* the samples) onto the PCs are called scores, while the importance of each original spectral variable in defining a certain PC is given by the loading coefficient. Both score and loading values can be represented in two-dimensional scatter plots. Simultaneous examination of the plots reveals that the information enclosed in the datasets is useful for characterizing the samples, according to the developmental stage. In more detail, in the score plot, it is possible to visualize groupings that indicate similarities among samples on the basis of the information derived from the mass spectra, and these can be associated with particular characteristics of the samples analyzed. Subsequently, an examination of both the loading plot and the score plot allows chemical characterization of the samples to be achieved, revealing which *m/z* peaks are the most important in defining each sub-set of samples under consideration. The relationship between the score and loading plots is evident from the co-directionality of objects and variables in related score and loading plots.

Linear Discriminant analysis

Linear discriminant analysis (LDA) was performed as supervised discriminant classification technique. Discriminant methods look for a delimiter that divides the global domain into a number of regions, each assigned to one of the classes. This delimiter identifies an open region for each class and such regions determine the assignment of the samples to one of the classes [8,9]. Model validation (*i.e.* evaluation of the predictive ability of the model) was performed by means of cross-validation (CV) [9]. For this study, 10 cross-validation deletion groups were selected, meaning that the all samples were divided 10 times systematically in a training set (objects used for building the classification model) and a test set (remaining objects used to evaluate the predictive ability of the model), with all the samples being in the evaluation set only once. Eventually, the final model was built with the objects all. In this study, LDA was applied on different datasets of TIC normalized mass spectra: 49 rows (samples) and 65001 variables (*m/z* values); 49 rows and 60001 variables; 49 rows and 20 variables (after DF-PCA, by selecting the PCs as new variables, instead of the original mass spectral data). Four classes were described: blastocysts produced *in vitro* (n= 13) and produced *in vivo* (n= 8) and immature oocytes (n= 13) and matured *in vitro* oocytes (n= 15). For the first two datasets, LDA was performed for evaluating separately the prediction ability of positive and negative ion mode mass spectra in classifying the samples, according to their developmental stage and *in vivo* vs. *in vitro* condition. Conversely, improving performances in classification ability, when considering positive and negative ion mode mass spectra together, were evaluated by applying LDA on the third dataset, deriving from the data fusion strategy. In this third LDA, for each CV deletion group, the preliminary PCA was performed just considering the training sample set. In more detail, 10 PCs (for each dataset) were computed each time with the training samples and used for building the classification model. The evaluation sample sets – samples that did not contribute in the model building process - were projected in the PC space and used to estimate the global prediction rate for all classes, so as the CV prediction rate for each class. This is a so-called complete validation strategy [9]. The CV prediction rate is the percentage of correct predictions on the objects in the CV evaluation sets. The CV confusion matrix shows how many samples belonging to a certain category were correctly/incorrectly assigned by the classification rule to that category. Indeed, in this matrix, each element gives the number of samples of the row category assigned to the column category. When the matrix is diagonal (entries outside the main diagonal are all zero) there is a perfect prediction of all the samples.

RNA extraction and quantitative RT-PCR

Poly (A)+mRNA extraction was performed using the Dynabeads® mRNA DIRECT Kit (Invitrogen, Carlsbad, USA). Briefly, individual oocytes or embryos were lysed by the addition of 40 μL of lysis /binding buffer (100 mM Tris-HCL pH 8.0, 500 mM LiCL, 10 mM EDTA, 1 % lithium dodecyl sulfate, 5 mM dithiothreitol) and incubated at room temperature for 10 min. After that, 1 pg of rabbit globin RNA (BRL, Gaithersburg, USA) was added to each preparation as an exogenous standard [10-12]. Five μL of prepared Dynabeads Oligo d(T)25 were added to the lysate and incubated at 25 °C on a shaker to allow binding for 15 min. The beads with the bound poly(A)+ mRNA were then separated using a Dynal MPC-E-1 magnetic separator. The beads and mRNA were washed with two different buffers (A and B) according to the manufacturers’ manual. In the final step, the mRNA was eluted from the beads by incubation in 11 μL of sterile water for 2.5 min at 68 °C and the mRNA was immediately used for reverse transcription. Reverse transcription (RT) was performed in a total volume of 20 μL consisting of 2 μL of 10x RT buffer (Invitrogen, Carlsbad, USA), 2 μL 50 mM MgCL2 (Invitrogen, Carlsbad, USA), 2 μL 10 mM dNTP solution (Amersham Biosciences, Piscataway, USA), 1 μL (20 units) of RNAsin (Applied Biosystems, Darmstadt, Germany), 1 μL (50 units) murine leukemia virus (MuLV) reverse transcriptase (Applied Biosystems, Darmstadt, Germany), and 1 μL hexamers (50 μM) (Applied Biosystems, Darmstadt, Germany), and 11 μL mRNA of target samples. The samples were incubated at 25 °C for 10 min for annealing and then incubated at 42 °C for 1 h in a thermocyler. Finally, samples were heated to 95 °C for 5 min. Quantitative Real-time PCR (qRT-PCR) was performed in 96-Well Optical Reaction Plates (Applied Biosystems, Darmstadt, Germany). The PCR mix, in each well, included 10 μL 2X Power SYBR Green PCR Master Mix (Applied Biosystems, Darmstadt, Germany), 7.2 μL dH2O, 0.4 μL each of the forward and reverse primers (5 μM) in a final reaction volume of 20 μL. Primer sequences of the target genes are summarized in Table A. Rabbit globin (50 fg) was amplified in parallel with the target genes for normalization. The PCR reaction was carried out in an ABI 7500 Fast Real-Time System (Applied Biosystems, Darmstadt, Germany) using the following program: denaturation and activation of the Taq Polymerase for 10 min at 95 °C, followed by 40 cycles at 95 °C for 15 sec and 60 °C for 1 min and a final slow heating cycle to obtain dissociation curves. A cDNA standard dilution of pooled immature oocytes was included on every plate to generate standard curves for each target gene. Data were processed using the Sequence Detection Software 1.3.1 (Applied Biosystems, Darmstadt, Germany). Relative mRNA concentration of each gene was calculated by the standard curve method. Normalization of the results obtained for each gene was performed by calculating the ratio to the level of globin mRNA.

**Table A.** **List of primers pairs used for quantitative PCR.**

| **Genes** | **Primer (Position)** | **Sequences (5´- 3´)** | **Amplicon length (bp)** | **Accession no.** | **Reference** |
| --- | --- | --- | --- | --- | --- |
| **ACAT1** | fw (906-930)  rev (949-972) | GCAGCTAAGAGGCTCAATGTTAAAC  GTTCTACAGCAGCATCAGCAAATG | 67 | NM_001046075 | − |
| **CPT1b** | fw (1158-1177)  rev (1214-1233) | CCCAGGGAAGGACACAGAGT  AAATCGGCCCTTGTGGTAGA | 76 | NM_001034349.2 | − |
| **FASN** | fw (6418-6438)  rev (6496-6515) | AGCTTTGTGTTGGCAGAGAAG  AGTCACGGATGCCCAGGAT | 97 | NM_001012669 | − |
| **SREBP1** | fw (143-162)  rev (195-209) | CCAGCTGACAGCTCCATTGA  TGCGCGCCACAAGGA | 67 | AW462480 | [[13](#_ENREF_13)] |
| **SCAP** | fw (332-349)  rev (482-501) | GGTCACTTTCCGGGATGG  TGGGTAGCAGCAGGCTAAGA | 169 | NM_001101889.1 | [[14](#_ENREF_14)] |
| **Globin** | fw (241-260)  rev (548-569) | GCAGCCACGGTGGCGAGTAT  GTGGGACAGGAGCTTGAAAT | 256 | X04751 | [[15](#_ENREF_15)] |

**References**

1. Racedo SE, Wrenzycki C, Herrmann D, Salamone D, Niemann H (2008) Effects of follicle size and stages of maturation on mRNA expression in bovine in vitro matured oocytes. Mol Reprod Dev 75: 17-25.

2. Parrish JJ, Susko-Parrish JL, Leibfried-Rutledge ML, Critser ES, Eyestone WH et al. (1986) Bovine in vitro fertilization with frozen-thawed semen. Theriogenology 25: 591-600.

3. Parrish JJ, Susko-Parrish JL, Winer WA, First NL (1988) Capacitation of bovine sperm by heparin. Biol Reprod 38: 1171-1180.

4. Wrenzycki C, Herrmann D, Keskintepe L, Martins A, Sirisathien S et al. (2001) Effects of culture system and protein supplementation on mRNA expression in pre-implantation bovine embryos. Hum Reprod 16: 893-901.

5. Oropeza A, Wrenzycki C, Herrmann D, Hadeler KG, Niemann H (2004) Improvement of the developmental capacity of oocytes from prepubertal cattle by intraovarian insulin-like growth factor-I application. Biol Reprod 70: 1634-1643.

6. Wrenzycki C, Lucas-Hahn A, Herrmann D, Lemme E, Korsawe K et al. (2002) In vitro production and nuclear transfer affect dosage compensation of the X-linked gene transcripts G6PD, PGK, and Xist in preimplantation bovine embryos. Biol Reprod 66: 127-134.

7. Ferreira CR, Eberlin LS, Hallett JE, Cooks RG (2012) Single oocyte and single embryo lipid analysis by desorption electrospray ionization mass spectrometry. J Mass Spectrom 47: 29-33.

8. Oliveri P, Downey G (2012) Multivariate class modeling for the verification of food-authenticity claims. Trends Anal Chem 35: 74-86.

9. Forina M, Oliveri P, Casale M (2010) Complete validation for classification and class modeling procedures with selection of variables and/or with additional computed variables. Chemometr Intell Lab Syst 102: 110-122.

10. Niemann H, Carnwath JW, Herrmann D, Wieczorek G, Lemme E et al. (2010) DNA methylation patterns reflect epigenetic reprogramming in bovine embryos. Cell Reprogram 12: 33-42.

11. Heinzmann J, Hansmann T, Herrmann D, Wrenzycki C, Zechner U et al. (2011) Epigenetic profile of developmentally important genes in bovine oocytes. Mol Reprod Dev 78: 188-201.

12. Diederich M, Hansmann T, Heinzmann J, Barg-Kues B, Herrmann D et al. (2012) DNA methylation and mRNA expression profiles in bovine oocytes derived from prepubertal and adult donors. Reproduction 144: 319-330.

13. Loor JJ, Dann HM, Everts RE, Oliveira R, Green CA et al. (2005) Temporal gene expression profiling of liver from periparturient dairy cows reveals complex adaptive mechanisms in hepatic function. Physiol Genomics 23: 217-226.

14. Viturro E, Koenning M, Kroemer A, Schlamberger G, Wiedemann S et al. (2009) Cholesterol synthesis in the lactating cow: Induced expression of candidate genes. J Steroid Biochem Mol Bio 115: 62-67.

15. Cheng JF, Raid L, Hardison RC (1986) Isolation and nucleotide sequence of the rabbit globin gene cluster psi zeta-alpha 1-psi alpha. Absence of a pair of alpha-globin genes evolving in concert. J Biol Chem 261: 839-848.
