# Supplementary figures and images for: Desorption Electrospray Ionization Mass Spectrometry Reveals Lipid Metabolism of Individual Oocytes and Embryos
Source: PLoS One. 2013 Sep 20;8(9):e74981. doi: 10.1371/journal.pone.0074981 (PMC3779253; doi:10.1371/journal.pone.0074981)

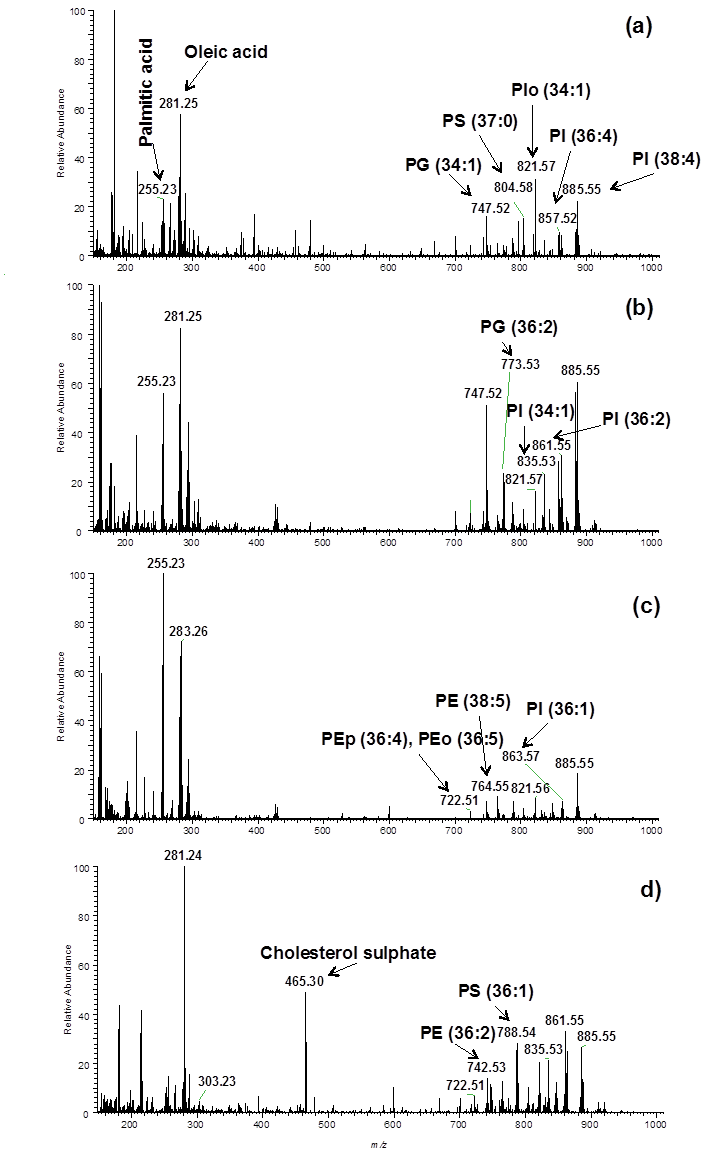

Supplement: Figure S1 — Representative high resolution DESI-MS mass spectra in the negative ion mode. (a) immature oocyte; (b) in vitro matured oocyte; (c) blastocyst produced in vitro; (d) blastocyst produced in vivo. See text and Table S2 in File S1 for tentative lipid class assignments of the major peaks. (TIF) [file pone.0074981.s003.tif]

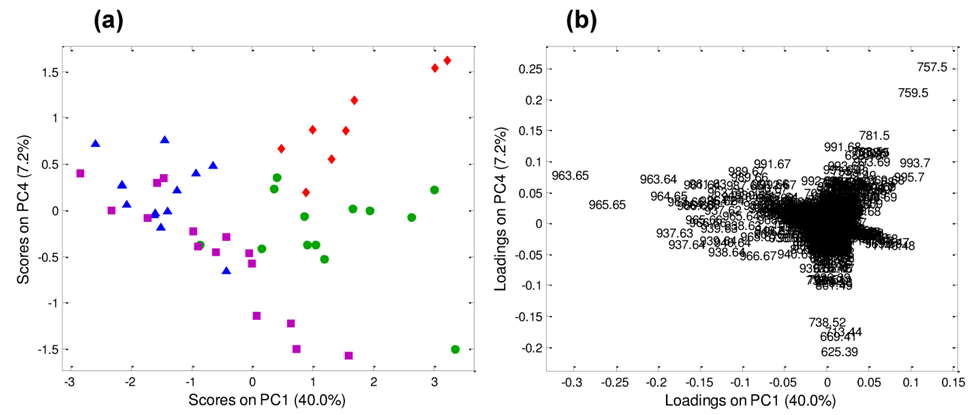

Supplement: Figure S2 — PCA of the positive ion mode mass spectral data. Blastocysts in vitro (green circles, n=13), blastocysts in vivo (red diamonds, n=8), immature oocytes (blue triangles, n=13) and in vitro matured oocytes (violet squares, n=15). (a) PC1 vs. PC4 score plot. (b) PC1 vs. PC4 loading plot labeled in terms of m/z ratio. (TIF) [file pone.0074981.s004.tif]

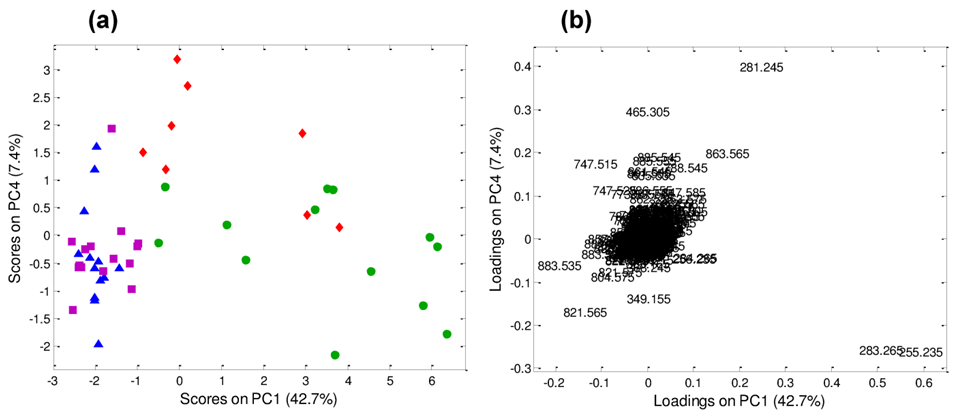

Supplement: Figure S3 — PCA of the negative ion mode mass spectral data. In vitro blastocysts (green circles, n=13), in vivo blastocysts (red diamonds, n=8), immature oocytes (blue triangles, n=13) and in vitro matured oocytes (violet squares, n=15). (a) PC1 vs. PC4 score plot. (b) PC1 vs. PC4 loading plot labeled in terms of m/z ratio. (TIF) [file pone.0074981.s005.tif]

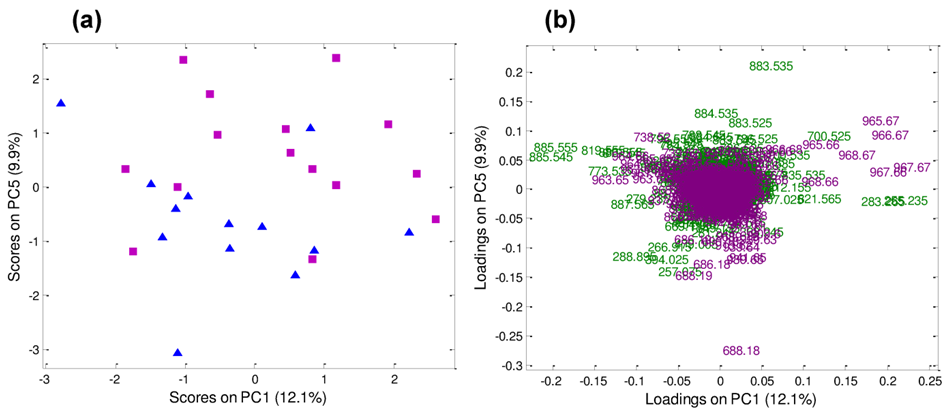

Supplement: Figure S4 — DF-PCA considering only oocytes. (a) PC1 vs. PC5 score plot. Blue triangles: immature oocytes (n=13); violet squares: in vitro matured oocytes (n= 15). (b) PC1 vs. PC5 loading plot labeled in terms of m/z ratio (green: negative ion mode; violet: positive ion mode). (TIF) [file pone.0074981.s006.tif]
